# Coordinated Cut and Bypass: Replication of Interstrand Crosslink-Containing DNA

**DOI:** 10.3389/fcell.2021.699966

**Published:** 2021-06-28

**Authors:** Qiuzhen Li, Kata Dudás, Gabriella Tick, Lajos Haracska

**Affiliations:** ^1^HCEMM-BRC Mutagenesis and Carcinogenesis Research Group, Institute of Genetics, Biological Research Centre, Szeged, Hungary; ^2^Mutagenesis and Carcinogenesis Research Group, Institute of Genetics, Biological Research Centre, Szeged, Hungary

**Keywords:** interstrand crosslink, DNA repair, translesion synthesis polymerases, PCNA ubiquitylation, structure-specific nuclease

## Abstract

DNA interstrand crosslinks (ICLs) are covalently bound DNA lesions, which are commonly induced by chemotherapeutic drugs, such as cisplatin and mitomycin C or endogenous byproducts of metabolic processes. This type of DNA lesion can block ongoing RNA transcription and DNA replication and thus cause genome instability and cancer. Several cellular defense mechanism, such as the Fanconi anemia pathway have developed to ensure accurate repair and DNA replication when ICLs are present. Various structure-specific nucleases and translesion synthesis (TLS) polymerases have come into focus in relation to ICL bypass. Current models propose that a structure-specific nuclease incision is needed to unhook the ICL from the replication fork, followed by the activity of a low-fidelity TLS polymerase enabling replication through the unhooked ICL adduct. This review focuses on how, in parallel with the Fanconi anemia pathway, PCNA interactions and ICL-induced PCNA ubiquitylation regulate the recruitment, substrate specificity, activity, and coordinated action of certain nucleases and TLS polymerases in the execution of stalled replication fork rescue via ICL bypass.

## Introduction

Our genome is constantly exposed to different exogenous and endogenous DNA damaging factors. Chemotherapeutic drugs, such as cisplatin or mitomycin C and metabolites like those from lipid peroxidation can cause interstrand crosslinks (ICLs), covalent links between the opposite strands of the DNA (reviewed in Stone et al., 2008). ICLs prevent strand separation, physically blocking replication and transcription. Stalled replication forks may collapse, causing DNA double-strand breaks, which can lead to chromosomal rearrangements, carcinogenesis, or cell death (reviewed in Negrini et al., 2010; Lenart and Krejci, 2016). ICLs have significant clinical relevance; inactivation of the Fanconi Anemia (FA) ICL repair pathway leads to FA. Patients diagnosed with FA suffer from progressive bone marrow failure and have a higher risk of developing cancer (reviewed in Shimamura and Alter, 2010). Due to their high cytotoxicity, ICL-inducing agents are the earliest and most commonly applied chemotherapeutic drugs (Rosenberg et al., 1969).

Since ICLs pose a high risk to cell survival and genome integrity, cells have developed multiple pathways to repair this type of DNA lesion. Nucleotide excision repair (NER) is able to recognize and remove ICL lesions in non-S-phase cells as well, while when ICLs block ongoing replication forks, in higher eukaryotic cells, the Fanconi anemia complementation group (FANC) DNA repair proteins that belong to the Fanconi Anemia (FA) pathway are believed to be the main operating defense system (reviewed in Wood, 2010). Activation of the FA pathway leads to the recruitment of structure-specific nucleases and translesion DNA synthesis (TLS) polymerases to enable the bypass of the lesion as well as facilitate the recombination-dependent rescue system (Howlett et al., 2002; Sarkar et al., 2006; Hicks et al., 2010; Kim and D’Andrea, 2012). However, a general defense system, the so-called Rad6-18 postreplication repair system, also comes into play when replication encounters unrepaired DNA damage, such as ICLs (Shen et al., 2006). Rad6-Rad18-dependent monoubiquitination of proliferating cell nuclear antigen (PCNA) initiates a number of subsequent replication fork rescue processes and is believed to serve as a key regulatory step in stalled replication fork rescue (reviewed in Chang and Cimprich, 2009).

Although the FA as well as the Rad6-Rad18 postreplication repair pathway become activated when replication stalls at ICLs, their interplay has been less characterized. In this review, we are placing the focus on sensors of ICLs, nucleases for ICL unhooking, and TLS polymerases for ICL adduct bypass with particular emphasis on parallels and possible interplays between their regulation by ubiquitylation of the FANCI and FANCD2 heterodimer (ID2) and ubiquitylation of PCNA during the rescue of replication forks stalled at ICLs.

## Sensors and Transducers of ICL Repair Pathways

ICL repair is mostly activated during the S phase, but there are secondary mechanisms that are active in quiescent cells as well (Williams et al., 2012). Although these pathways have distinct mechanisms depending on the cell cycle, they all have common key steps. First, the lesion is recognized by sensor proteins that recruit other downstream regulators. During the G0/G1 phase, mainly nucleotide excision repair (NER) pathways monitor the genome, searching for ICL-caused distortion in the DNA. The XPA (Xeroderma pigmentosum A) and RPA (replicative protein A) protein, after having been recruited to the damaged area, load the structure-specific nuclease ERCC1-XPF onto the DNA (Volker et al., 2001; Tsodikov et al., 2007).

**FIGURE 1:**
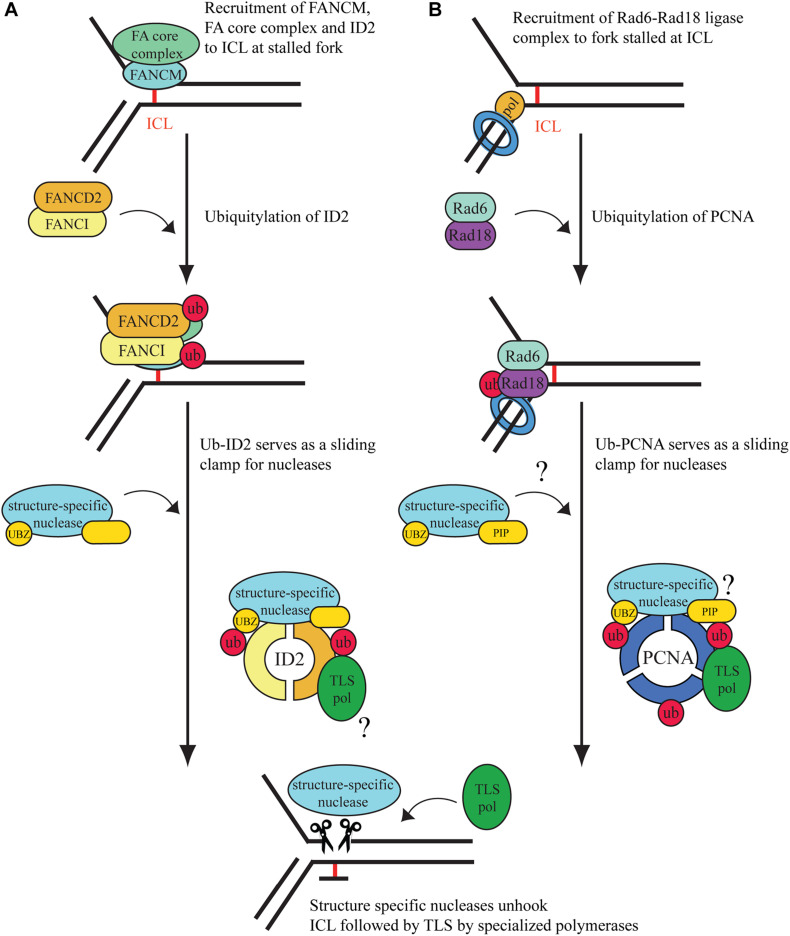
Interstrand crosslink repair pathways at the stalled replication fork. **(A)** Replication-dependent ICL repair is considered to be carried out mainly by the Fanconi anemia pathway. After damage recognition, the ID2 complex is ubiquitylated by the activated FA core complex. The monoubiquitinated ID2 complex can further recruit structure-specific nucleases for unhooking. Gap filling is carried out by TLS polymerases, but the mechanism behind the recruitment of the polymerases is yet unknown. We hypothesize that Ub-ID2 serves as a DNA- encircling sliding clamp for TLS polymerases and nucleases. **(B)** When the ongoing DNA replication fork is blocked by DNA lesions, such as ICLs, upstream factors recognize it, and PCNA is monoubiquitinated by RAD6/RAD18. We hypothesize that monoubiquitinated PCNA acts as a hub for the recruitment of certain PIP box- and UBZ domain-containing structure-specific nucleases to unhook the ICL lesion. Translesion synthesis polymerases are also recruited to the site of damage by Ub-PCNA, which complete the bypass of the DNA damage. The covalent bond of the monoadduct is removed by nucleotide excision repair.

In the S phase of the cell cycle, ICLs cause replication fork stalling followed by activation of Ataxia telangiectasia (ATR)-dependent damage signaling, which will prevent dormant replication fork firing, while stabilizing the stalled replication fork (Luke-Glaser et al., 2010; Schwab et al., 2010). As shown in Figure 1A, the binding of FANCM at the site of the ICL has an essential role since it provides a platform for anchoring other FA proteins (Collis et al., 2008; Deans and West, 2009). Although FANCM seems to be an upstream regulator of ATR, its activity is also induced by ATR-dependent phosphorylation (Ciccia et al., 2007; Collis et al., 2008; Singh et al., 2013). When the phosphorylated FANCM recognizes ICLs, it recruits the FA core complex, which has a ubiquitin ligase activity transferring the ubiquitin with the help of FANCL, a RING-domain containing E3, from the UBE2T/FANCT, an E2 enzyme, to the FANCI and FANCD2 heterodimer (ID2) at lysines 523 and 561, respectively (Smogorzewska et al., 2007; Alpi et al., 2008; Huang et al., 2014). Prior to monoubiquitination, ID2 seems to be recruited by the FANC core complex to the damage where its DNA-binding generates the needed conformational change in FANCD2 for its ubiquitylation. Ubiquitylation closes the ID2 complex into a clamp conformation (Liang et al., 2015; Alcón et al., 2020; Tan et al., 2020). The activated ID2 complex then serves as a central hub for subsequent molecular events by enabling the recruitment of proteins that provide finally the rescue of the replication fork stalled at the ICL. These proteins include nucleases for unhooking ICLs, TLS polymerases for bypass of the unhooked adduct, repair factors for ICL elimination, and several factors of homologous recombination (Figure 1A).

Interestingly, in addition to the FANCM-dependent mechanism, other ICL-sensors, such as the Ubiquitin-Like PHD And RING Finger Domain-Containing Protein 1 (UHRF1) was also identified (Liang et al., 2015). UHRF1 and its paralog, UHRF2, can recruit FANCD2 to the site of the ICL and stimulate monoubiquitination of the ID2 complex (Motnenko et al., 2018).

## PCNA Ubiquitylation, a Sensor and Signal Transducer in the Replication of ICL Lesions

The homotrimer PCNA is the master regulator of the replication fork. PCNA forms a sliding clamp over the DNA double strand and serves as a processivity factor for replicative polymerases. Encountering an unrepaired DNA lesion, such as an ICL results in the stalling of the replicative polymerase, which leads to the recruitment of Rad6-Rad18, a ubiquitin conjugating-ligase protein complex, which facilitates PCNA monoubiquitination (Bailly et al., 1997; Yoon et al., 2012). Monoubiquitinated PCNA can recruit TLS polymerases, which synthesize DNA across the damaged region either in an error-free or an error-prone mode, depending on the actual lesion and the TLS polymerase accessed (reviewed in Prakash et al., 2005). Many TLS polymerases, such as Pol eta, Pol kappa, and Pol iota exhibit conserved PCNA-interacting (PIP) as well as ubiquitin-binding (UBD) domains, by which they can strongly associate with monoubiquitinated PCNA (Ub-PCNA), which provides their timely access to the primer ends at stalled forks when replication encounters a barrier (Haracska et al., 2001a; Plosky et al., 2006). The PIP and UBD domains are also exhibited in certain nucleases implicated in ICL repair, such as SNM1A and FAN1, which raises the possibility that, similarly to Ub-ID2, Ub-PCNA can also play a role in their targeting to replication-stalling ICLs (Yang et al., 2010; Porro et al., 2017). Monoubiquitinated PCNA can also undergo polyubiquitylation in an Mms2–Ubc13 (E2)- and HTLF-SHPRH (E3)-dependent manner (Unk et al., 2008). Polyubiquitylated PCNA can initiate template switching, an error-free pathway for stalled replication fork rescue, which can involve fork reversal. Fork reversal can place ICL back to a general double-stranded DNA region, making it accessible for excision repair pathways, such as NER. Interestingly, RAD18 also has a function in ICL repair, independent of ubiquitinated PCNA. RAD18 has been shown to indirectly regulate the ubiquitination and loading of FANCD2 and FANCI in S phase, which suggests a possible role for RAD18 as an E3 ligase of the FA core complex (Williams et al., 2011). Recently, the RING finger and WD repeat domain-containing protein 3 (RFWD3) has been identified as an activator of various rescue mechanisms at the stalled replication fork, such as translesion synthesis and homologous recombination (HR) (Elia et al., 2015; Inano et al., 2017). Importantly for ICL repair, RFWD3 was suggested to be a FA gene since its biallelic mutation was found in a child with FA, which was also supported by results in cellular systems and animal model (Knies et al., 2017). Indicating complex interplays, the interaction of RFWD3 and PCNA stabilizes RFWD3 to the replication fork, and PCNA also interacts with FANCM, pointing to its possible function in lesion recognition (Rohleder et al., 2016; Lin et al., 2018).

## Structure-Specific Nucleases as Executors of ICL Unhooking at the Stalled Replication Fork

**FIGURE 2:**
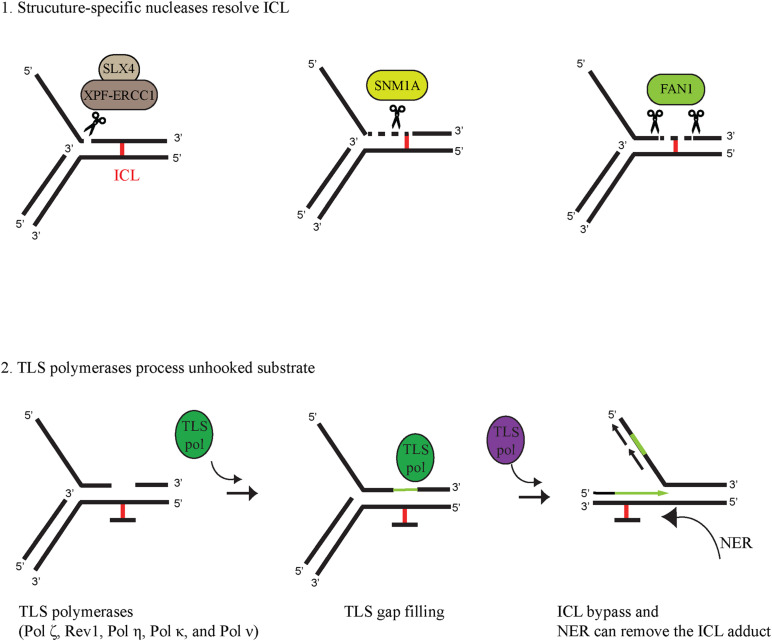
Coordinated activity of structure-specific nucleases and TLS polymerases in ICL bypass (1) Structure-specific nucleases unhook ICL-containing DNA. SLX4 serves as a scaffold protein for XPF-ERCC1 to digest the dsDNA phosphodiester bond at the 5′ primer of the junction between dsDNA and ssDNA. The digested substrate can be further processed by the 5′-3′ exonuclease activity of SNM1A. FAN1 can completely unhook the ICL-containing substrate due to its 5′-3′ endo/exonuclease activity. (2) TLS polymerases can process the unhooked ICL-containing DNA by filling in the gap generated by ICL-unhooking nucleases and by extending the primer through the unhooked ICL adduct. Finally, the ICL monoadduct can be removed by another DNA repair pathway, such as nucleotide excision repair (NER).

Following lesion recognition, the ICL is released from one of the DNA strands by a process called unhooking. During unhooking, structure-specific nucleases nick the DNA on both sides of the ICL, removing it from one of the parental strands. Several nucleases have been implicated in ICL unhooking, such as XPF-ERCC1, MUS81-EME1, SLX1, SNM1A, and FAN1 the deficiency of which renders cells sensitive to ICL-generating agents. These nucleases alone or in collaboration can cleave the DNA on both sites of the ICL, leaving a gap, which can be filled in subsequently by TLS polymerases (Figure 2). XPF and SLX1 nucleases are considered to incise at 3′- and 5′-sides of the ICL (Kuraoka et al., 2000; Fricke and Brill, 2003), respectively, while MUS81 cuts at 3′-sides in specific cases (Ciccia et al., 2003). Various interactions can modulate the cleavage specificity of these enzymes; the nuclease activity of SLX1 can be extremely enhanced by its interaction with SLX4 (Fricke and Brill, 2003). As a scaffold protein, SLX4 can interact with several other nucleases and via its UBZ-domain-mediated binding to Ub-ID2 it can recruit XPF-ERCC1-MUS81-EME1-SLX1-SLX4 to the ICL (Fekairi et al., 2009; Castor et al., 2013). Ub-ID2 might play a role in the recruitment of other nucleases, as well, as it was proposed for FAN1; however, here we put more focus on interaction of SNM1A and FAN1 with Ub-PCNA in the ICL repair process. The SNM1A nuclease contains a ubiquitin−binding zinc finger (UBZ) domain at the N-terminal and a PIP box in the middle region of the protein (Yang et al., 2010). SNM1A has an intrinsic 5′ to 3′ exonuclease activity and was shown to be epistatic with the XPF-ERCC1 endonuclease that can nick the DNA 5′ from the ICL (Wang et al., 2011). Their coordinated action in ICL unhooking was shown; the XPF-ERCC1-generated nick provided an entry point for SNM1A exonuclease activity (Wang et al., 2011). Recently, SNM1A has been shown to have single-strand-specific endonuclease activity as well (5′ and 3′overhangs, hairpins, flaps, and gapped substrates) (Buzon et al., 2018).

The FAN1 nuclease also possesses a UBZ domain and a PIP box at the N-terminal end (Smogorzewska et al., 2007; Pennell et al., 2014). Originally, several reports described FAN1 as a member of the FA pathway since its action on ICL-containing DNA was described to be dependent on monoubiquitinated FANCD2 (Kratz et al., 2010; Liu et al., 2010; MacKay et al., 2010). However, later on, it turned out that patients with biallelic FAN1 mutations do not develop FA, and FAN1 does not show epistasis with other FA genes, which indicates some other role for FAN1 in ICL repair (Zhou et al., 2012; Lachaud et al., 2016b). FAN1 has an endonuclease activity and is able to incise 5′ to the ICL at the 4th nucleotide after the replication fork junction on the 5′ flap model substrate (Figure 2). FAN1 has a 5′→ 3′ exonuclease activity as well, initiating cleavage 4 nt from the 5′ end on single- and double-stranded DNA (MacKay et al., 2010; Lachaud et al., 2016a). FAN1 is able to unhook nitrogen mustard-induced interstrand crosslinks *in vitro* due to its nuclease activity (Pizzolato et al., 2015). FAN1 also interacts with ubiquitin-PCNA and enhances PCNA ubiquitylation after mitomycin C treatment (Porro et al., 2017).

## TLS Polymerases Process Substrates Unhooked by Nucleases

Replicative polymerases, such as the human Pol δ and Pol ε have high fidelity and possess 3′→ 5′ exonuclease activity as a proofreading function to ensure precise DNA replication (Johnson et al., 2015). However, there is a cost of high fidelity since DNA contains many lesions, such as base adducts, photoproducts, intrastrand and interstrand crosslinks, which cannot be accommodated by the tight active sites of the replicative polymerases, leading to blocked replication fork machinery (Bezalel-Buch et al., 2020). Stalling of replication can lead to strand breaks, chromosomal rearrangement, and other genome-destabilizing events; to avoid this, the so-called DNA damage tolerance (DDT) pathways, such as the Rad6-Rad18-dependent PCNA-monoubiquitination-mediated one come into play upon fork stalling (Davies et al., 2008). One sub-branch of these DDT pathways is translesion synthesis, in which, at the damage, low-fidelity polymerases take over the 3′ primer end from the replicative polymerase and insert either the correct or uncorrect nucleotide opposite the lesion, leading to error-free or error-prone bypass. As shown in Figure 2, bypass of ICL may involve TLS polymerase action at two points; in the gap filling after the nuclease has unhooked the ICL and in the replication through the adduct. Based on genetic assays with TLS polymerase-deficient cells as well as biochemical findings, many TLS polymerases have been implicated in the bypass of the unhooked ICL (Ho et al., 2011; Roy et al., 2016; Bezalel-Buch et al., 2020). Sensitivity assays performed by treating cells with various crosslinking agents revealed ICL-repair functions for Pol ζ, Rev1, Pol η, Pol κ, and Pol ν (reviewed in Ho and Schärer (2010). Although various crosslinking agents can produce a wide variety of ICLs requiring different TLS polymerases for bypass, based on cisplatin and mitomycin C exposure, REV3-encoded Pol ζ and REV1 are believed to be among the main players of ICL bypass (Hicks et al., 2010). Genetic evidence also supports the involvement of Pol η in a more general role in ICL bypass, while Pol κ seems to be more restricted to minor groove DNA adducts and Pol ν to major groove ICL bypass (Zhang et al., 2000; Acharya et al., 2008; Yamanaka et al., 2010; Roy et al., 2016). TLS polymerases can bypass DNA lesions in a two-step fashion, first inserting a nucleotide opposite the lesion and then extending opposite from the lesion (Johnson et al., 2000; Haracska et al., 2001b). Certain TLS polymerases can carry out both steps, but often it requires the collaboration of two polymerases: an inserter and an extender (reviewed in Ho and Schärer, 2010). Even though the deficiency of a certain TLS polymerase does not cause strong hypersensitivity to crosslinking agents, its involvement in ICL bypass cannot be ruled out because cells can use multiple TLS polymerases as inserters as well as extenders. Although purified REV1 together with Pol ζ show complete bypass synthesis, experiments using Xenopus egg extracts indicate that Pol ζ and REV1 are required only for the extension step past a cisplatin-induced ICL (Bezalel-Buch et al., 2020). Pol η alone is able to carry out both the insertion and the extension steps across various major ICL lesions (Roy et al., 2016). Pol η and many other TLS polymerases, such as Pol κ and Pol ι contain PIP and ubiquitin-binding motif (UBM)/UBZ domains allowing them to interact with Ub-PCNA, which can target them to the site of the stalled fork at the ICL as well as stimulate their synthetic activity (Haracska et al., 2001a; Plosky et al., 2006).

## Discussion

Cytotoxic ICL lesions pose a considerable threat to cells regardless of cell phase. Cells can remove ICLs by NER during the G0/G1 phase, but some may escape repair and cause stalling of the replication machinery. In higher eukaryotic cells, the FA pathway is considered the main defense system to repair ICLs during the S phase of the cell cycle. In this review, we compare the FA pathway to a more general defense system, the Rad6-RAD18-dependent PCNA monoubiquitination, which is activated when replication stalls at various lesions, including ICLs. We also point out the similarities between the FA and Rad6-Rad18 pathways in dealing with the ICL at the stalled replication fork (Figure 1) and summarize our current knowledge on ICL-unhooking nucleases and ICL bypass polymerases (Figure 2), and reach the following conclusions. First, when replication encounters an ICL, monoubiquitation of the central hub proteins, ID2 and PCNA, is a critical step for the operation of the FA and Rad6-Rad18 pathways, respectively. Second, several proteins implicated in ICL repair can interact with Ub-ID2 as well as Ub-PCNA, such as the FAN1 nuclease, which exhibits PIP and UBZ domains for timely binding to the stalled fork (Buzon et al., 2018). Interestingly, deficiency of FAN1 can be partially complemented by the SNM1A nuclease, which also exhibits PIP and UBZ domains (Yang et al., 2010; Buzon et al., 2018). Third, ubiquitylation can provide access to unhooked ICL adduct bypass of various TLS polymerases exhibiting PIP and, in most cases, UBZ domains as well, which indicates similarities between the recruitment of nucleases and polymerases. Fourth, after completion of the repair process, both FA and Rad6-Rad18 DDT pathways are terminated by USP1-dependent deubiquitylation of their central hub proteins (Nijman et al., 2005; Huang et al., 2006). Importantly, PCNA encircles DNA as a homotrimeric sliding clamp, and each of its subunit can be ubiquitylated, providing three binding surfaces for PIP- and UBZ-domain-containing proteins. Thus, it is possible that proteins exhibiting these two domains, such as an ICL-unhooking nuclease like FAN1 and a TLS polymerase like Pol η, can bind to a Ub-PCNA ring at the same time, which would provide a high degree of coordination between ICL unhooking and bypass. Interestingly, recent structural studies revealed that ubiquitylation of the ID2 complex results in its conformational change which converts the ID2 to a clamp encircling the DNA (Alcón et al., 2020; Wang et al., 2020). The structural analogy between Ub-PCNA and Ub-ID2 is tempting and forces one to speculate whether Ub-ID2 can serve as a sliding clamp for TLS polymerases in ICL bypass. Also, it would be interesting to explore whether the Ub-PCNA and Ub-ID2 sliding clamps can bind ICL nucleases and TLS polymerases at the same time for efficient bypass. Finally, it remains to be explored whether Ub-PCNA and Ub-ID2 rings can interact and provide a joint sliding clamp for protein exchange and higher coordination between the FA and Ub-PCNA ICL damage bypass pathways.

## Author Contributions

QL and KD reviewed the literature and wrote the first draft of the manuscript. GT and LH revised and edited the manuscript. All authors approved the final version of the manuscript for submission.

## Conflict of Interest

The authors declare that the research was conducted in the absence of any commercial or financial relationships that could be construed as a potential conflict of interest.
